# C1QBP regulates mitochondrial plasticity to impact tumor progression and antitumor immune response

**DOI:** 10.3389/fphys.2022.1012112

**Published:** 2022-11-17

**Authors:** Qiping Wang, Dafei Chai, Navid Sobhani, Nan Sun, Praveen Neeli, Junnian Zheng, Hui Tian

**Affiliations:** ^1^ Jiangyin Clinical Medical College, Jiangsu University, Jiangyin, Jiangsu, China; ^2^ Department of Medicine, Baylor College of Medicine, Houston, TX, United States; ^3^ Cancer Institute, Xuzhou Medical University, Xuzhou, Jiangsu, China; ^4^ Center of Clinical Oncology, Affiliated Hospital of Xuzhou Medical University, Xuzhou, Jiangsu, China; ^5^ Jiangsu Center for the Collaboration and Innovation of Cancer Biotherapy, Cancer Institute, Xuzhou Medical University, Xuzhou, Jiangsu, China

**Keywords:** C1QBP, mitochondrial plasticity, metabolic flexibility, tumor progression, antitumor immune response, immunotherapy

## Abstract

Mitochondrial plasticity including mitochondrial dynamics, metabolic flexibility, and mitochondrial quality control, impact tumor cells’ progression and determine immune cells’ fate. Complement C1q binding protein (C1QBP) plays an indispensable role through regulating mitochondrial morphology, metabolism, and autophagy. C1QBP promotes mitochondrial plasticity to impact tumor metastasis and their therapeutic response. At the same time, C1QBP is involved in regulating immune cells’ maturation, differentiation, and effector function through the enhancement of mitochondrial function. In this regard, manipulation of C1QBP has been shown to adjust the competitive balance between tumor cells and immune cells. In the course of evolution, mitochondrial plasticity has endowed numerous advantages against the relentless microenvironment of tumors. In this current review, we summarize the current knowledge of the mechanism of C1QBP regulation of cancer and immunity. We explain this process in vision of potentially new anticancer therapies.

## Introduction

Mitochondria can rapidly sense energy availability and generate precursors necessary for the biosynthesis of amino acids, lipids, and nucleotides to meet cellular requirements or respond to adverse cellular stimulation (Mishra and Chan, 2016; Ahola et al., 2019). Most importantly, mitochondria function as the hub of numerous signal pathways, including the tricarboxylic acid (TCA) cycle, oxidative phosphorylation (OXPHOS), and fatty acid oxidation (FAO), which endow cells with the considerable adaptation to face a variety of challenging stresses (Vyas et al., 2016; Schvartzman et al., 2018).

Complement C1q binding protein (C1QBP), also known as p32 or hyaluronic acid-binding protein (HABP1), is a multicompartmental protein. In other words, C1QBP exists in multiple subcellular compartments, including the nucleus, endoplasmic reticulum, Golgi, and on the cell surface (Ghebrehiwet et al., 2001; Fogal et al., 2008). For example, C1QBP located on the cell surface interacts with tumor homing peptide, Lyp-1, which specifically recognizes an epitope in tumor cells (Fogal et al., 2008). Moreover, it binds with the pre-mRNA splicing factor (SF2/ASF) in the nucleus, which controls RNA splicing by sequestering an essential RNA splicing factor into an inhibitory complex (Petersen-Mahrt et al., 1999). However, increasing evidence has demonstrated that C1QBP predominantly resides in the mitochondria through its 33-residue N-terminal mitochondria-targeting signal (MTS) sequence and is essential for embryonic development, which is dependent on mitochondrial translation and OXPHOS (Muta et al., 1997; Fogal et al., 2010; Yagi et al., 2012; Zhai et al., 2021). It has been reported that *C1qbp* deletion induces embryos’ mid-gestation lethality and triggers severe dysfunction of the mitochondrial respiratory chain due to failure of mitochondrial protein synthesis. The association of C1QBP with mitochondrial RNA and mitochondrial ribosomes is required for some mitochondrial protein translation. Besides, C1QBP is also involved in mitochondrial autophagy to remove the dysfunctional mitochondria to maintain healthy cellular homeostasis (Jiao et al., 2015; Jiao and You, 2016). Consequently, C1QBP is integral to mitochondrial fitness, cell metabolism, and survival.

Given that tumor cells and T cells share the metabolic similarities of proliferation, expansion as well as activation, manipulation of mitochondrial plasticity would impact the metabolic competition between tumor cells and T cells in the context of the tumor microenvironment (TME). Tumor cells depend on C1QBP for progression and therapeutic resistance, while immune cells also depend on it for a persistent and powerful antitumor response. This review discusses the dual roles of C1QBP for mitochondrial function in tumor malignancy and immune cells. Finally, we give our expert opinion on potential new mitochondrial-dependent immune stimulation mechanism based-therapies targeting C1QBP.

## Complement C1q binding protein modulation of mitochondrial plasticity

Mitochondria are highly dynamic organelles constantly undergoing fission and fusion. When they are too damaged, they go through mitophagy, which is the selective degradation of mitochondria by autophagy. It has been elucidated that C1QBP plays a vital role in maintaining mitochondrial homeostasis through regulating mitochondrial morphology, metabolism, and mitophagy. Thereby this protein is fundamental for modulating mitochondrial plasticity and fitness.

### Complement C1q binding protein regulates mitochondrial morphology

Mitochondria undergo constant fission and fusion cycles according to the cellular bioenergetic situation. This fine-tuning of mitochondria is vital to give cells the optimal energy supply and survival. The mitochondrial architecture principally consists of optical atrophy 1 (OPA1), mitofusin 1/2 (Mfn1/2), the mitochondrial fission controlled by dynamin-related protein 1 (Drp1), and its mitochondrial adaptor fission protein 1 (Fis1) (Santel and Fuller, 2001; Chen and Chan, 2005; Loson et al., 2013).

C1QBP modulates mitochondrial morphology and integrity. *C1qbp* silencing not only promoted mitochondrial fragmentation but also repressed mitochondrial fusion (Fogal et al., 2010; Li et al., 2011; Hu et al., 2013; Noh et al., 2020). For example, Solhee Noh et al. found that genetic ablation of *C1QBP* activates the overlapping activity with m-AAA protease (OMA1) to cleave OPA1, which leads to mitochondrial fragmentation and swelling (Noh et al., 2020). C1QBP regulated mitochondrial morphology by regulating OMA1‐dependent proteolytic processing of OPA1. On the other hand, MengJie Hu et al. revealed that overexpression of C1QBP increased mitochondrial fibrils (Hu et al., 2013). At the same time, C1QBP siRNA-treated cells contained predominantly small spherical mitochondria compared with the mainly normal elongated mitochondria in control cells. To further explore the underlying mechanism, they examined the expression of *Mfn1, Mfn2,* and mitochondrial *Drp-1* and found that the C1QBP siRNA treatment resulted in a detectable loss of *Mfn1* and *Mfn2* and decreased Drp-1 levels. Here, siRNA-mediated *C1qbp* knockdown induced a more punctate mitochondrial morphology, which was associated with the reduction of the mitochondrial fusion mediator proteins, a sign of potential subsequent degradation.

### Complement C1q binding protein is involved in mitochondrial metabolism

Some tumor cells significantly depend on mitochondrial-mediated oxidative phosphorylation (Funes et al., 2007; Moreno-Sanchez et al., 2007). Since oxidative metabolism may recycle lactate, mitochondrial metabolism is advantageous for tumor cells that are highly glycolytic (Sonveaux et al., 2008). Moreover, glycolytic pathway oncogenes may upregulate some genes encoding mitochondrial proteins or those involved in mitochondrial biogenesis (Li et al., 2005). Additionally, mitochondria regulate cellular bioenergetic and biosynthetic processes of important cancer signaling pathways, such as proliferation, metastasis and self-renewal (Viale et al., 2014; Liu and Shi, 2020). Collectively, these findings suggest that oncogene-driven glycolytic metabolism should be balanced, at least in part, by concomitant changes to mitochondria. Mitochondrial metabolism may potentiate cellular plasticity and metabolic flexibility to deal with the relentless microenvironment.

C1QBP plays an indispensable role in mitochondrial function and metabolic regulation. For example, *C1qbp*-deficient mouse embryonic fibroblasts (MEFs) cells exhibit reduced OXPHOS function (Yagi et al., 2012). A mutation of *C1qbp* has also been suggested as a cause of mitochondrial respiratory chain disorder. In this regard, C1QBP is a crucial regulator of mitochondria-mediated OXPHOS.

### Complement C1q binding protein is responsible for mitochondrial quality control

A functional mitochondrial network responding to physiological adaptations and stress conditions is critical for a healthy cellular condition. Given that mitochondria are exposed to high levels of reactive oxygen species, they have evolved multiple systems of quality control to ensure that the requisite number of functional mitochondria are present to meet cellular demands. Mitochondria eliminate the damaged mitochondrial proteins through autophagy. Thus, understanding how C1QBP orchestrates mitophagy to maintain mitochondrial homeostasis could provide critical insights.

Jiao et al. (2015) reported that C1QBP controlled mitochondrial autophagy to help cells adapt better to the challenging microenvironment (Jiao and You, 2016). Serine/threonine kinase Unc-51-like kinase-1 (ULK1) is crucial in inducing mitophagy. This protein is susceptible to proteasome-mediated degradation. C1QBP regulates ULK1 stability by forming a protein complex with ULK1. The interaction between ULK1 and C1QBP is indispensable to maintaining steady-state levels of ULK1, thereby preventing its polyubiquitylation. Moreover, it has been shown that mitophagy defects can be recovered by re-introducing ULK1 into C1QBP-deficient cells, which suggested that C1QBP protected mitophagy through the prevention of ULK1 degradation. Considering that mitochondrial homeostasis and oxidative phosphorylation might be mutually beneficial (Wrighton, 2013), mitochondrial quality control may serve as an important mechanism for maintaining cellular energy homeostasis. Therefore, C1QBP exerts a protective effect under starvation conditions by both inducing Ulk1-dependent autophagy and maintaining oxidative phosphorylation. In this regard, C1QBP-Ulk1-mitophagy axis may provide a survival advantage when nutrients are scarce, which ultimately promotes tumorigenesis.

Taken together, mitochondrial plasticity including mitochondrial dynamics, metabolism, and autophagy is tightly linked to mitochondrial structure, function and homeostasis. C1QBP overexpression promotes mitochondrial plasticity to endow tumor cells with proliferation and malignant progression. On the contrast, *C1qbp* knocking down dampens mitochondrial adaptation and metabolic flexibility, which is responsible for tumor repression.

## Complement C1q binding protein is implicated in tumor progression

C1QBP is expressed at high levels in a significant number of tumor types, including melanoma, colon, ovarian, gastric, prostate, brain, and breast, compared with their nonmalignant counterparts (Chen et al., 2009; Amamoto et al., 2011; Yu et al., 2013; Niu et al., 2015; Wang et al., 2015; Gao et al., 2016; Rousso-Noori et al., 2021; Sinha et al., 2021). Various studies involving patients have revealed that C1QBP is positively linked to the tumor stage and poor prognosis, as shown in Table 1.

**TABLE 1:** C1QBP overexpressed in a variety of tumor cells is implicated in tumor malignant progression and tumor therapeutic treatment.

C1QBP as a tumor biomarker	C1QBP and tumor progression	Tumor therapies targeting C1QBP
C1QBP as a prognostic marker is related to metastasis and poor prognosis in cancer patients (Chen et al., 2009; Niu et al., 2015; Wang et al., 2015)	C1QBP interacts with PKC to modulate in cancer cell chemotaxis (Zhang et al., 2013)	C1QBP as a new target can be utilized for precise delivery of imaging or therapeutic agents to tumors (Sanchez-Martin et al., 2011)
C1QBP as a tumor biomarker is tightly associated with the lymph node and peritoneal metastasis in epithelial ovarian cancer patients (Yu et al., 2013)	C1QBP is involved in tumor invasion and migration (Prakash et al., 2011)	C1QBP antibody inhibits growth factor stimulated lamellipodia formation, cell migration and focal adhesion kinase activation as well as prevents even angiogenesis (Kim et al., 2016)
C1QBP as a critical regulator for tumor progression of prostate cancer is positively correlated with pathological stage and relapse of the disease (Amamoto et al., 2011)	C1QBP promotes tumor metastasis by targeting EMT markers, and modulating TME (Sinha et al., 2021)	C1QBP as the receptor of a nanoparticle drug, CGKRK nanoworms, has been found to be effective in orthotopic glioblastoma and breast cancer (Agemy et al., 2013)
C1QBP as a tumor diagnostic marker is correlated with progression and poor prognosis of the gastric cancer patients (Gao et al., 2016)	C1QBP shifts tumor metabolism to OXPHOS leading to the enhanced tumorigenicity (Fogal et al., 2010)	A C1QBP binding peptide displays improved tumor penetration and increased efficacy in suppressing breast tumor growth *in vivo* (Hunt et al., 2017)
C1QBP as a tumor associated antigen (TAA) is overexpressed in clinic low- and high-grade gliomas (Rousso-Noori et al., 2021)	C1QBP impacts the lamellipodia formation and a concomitant decrease in FAK kinase, thus promoting tumor migration and tumorigenesis (Kim et al., 2011)	C1QBP-specific CAR T cells recognize and specifically eliminate C1QBP expressing glioma cells and tumor derived endothelial cells (Rousso-Noori et al., 2021)

As expected, high expression levels of C1QBP are inversely correlated with tumor patients’ prognosis, making C1QBP a valuable independent prognostic marker of outcomes in tumor patients. For example, upregulation of *C1qbp* mRNA has been detected in low and high-grade gliomas, especially recurrent glioblastoma (GBM) (Rousso-Noori et al., 2021). This protein significantly is associated with the extent of the disease progression. It might also serve as a tumor-associated antigen (TAA) in gliomas.

Furthermore, C1QBP modulates tumor proliferation and metastasis. Its expression level in breast cancer was closely linked with distant metastasis and tumor node metastasis (TNM) stages (Wang et al., 2015). X Zhang et al. (2013) reported that during metastasis, the C1QBP up-modulation was confined to metastatic islands, which could have significant implications if further experiments could show it can regulate tumor metastasis. On this note, the authors showed that C1QBP held the activity of protein kinase C and modulated EGF-induced cancer cell chemotaxis, which thus impacted tumor cell migration. In contrast, C1QBP silencing attenuated tumor progression and metastasis *in vivo* by modulating the tumor microenvironment through inhibiting tumor angiogenesis and macrophage infiltration (Sinha et al., 2021). Based on such premises, C1QBP can be a target of a novel therapeutic approaches in the regulation of tumor progression and metastasis. Additionally, C1QBP has been observed to adhere to the cell surface and interact with integrin αvβ3, a regulatory molecule of cell migration. C1QBP-integrin interaction results in transcriptional upregulation of MT1-MMP expression and finally MMP-2 activation, leading to enhanced tumorigenicity and cell migration in B16F10 melanoma cell line (Prakash et al., 2011). Besides, *C1qbp* knocking down exhibits the disruption of lamellipodia formation and a concomitant decrease in focal adhesion kinase (FAK) kinase and receptor kinase, inhibiting cellular migration and tumorigenesis (Kim et al., 2011).

On the other hand, increasing evidence has shown that C1QBP-mediated tumorigenesis is tightly associated with mitochondrial metabolism maintenance. Fogal et al. (2010) found that knocking down C1QBP expression in human cancer cells strongly shifted their metabolism from oxidative OXPHOS to glycolysis. C1QBP deficiency resulted in less *in vivo* tumorigenicity*,* probably due to the reduced synthesis of the mitochondrial-DNA-encoded OXPHOS polypeptides. Thus, C1QBP is implicated in mitochondrial protein translation and affects several electron transport complexes’ functions. On the other hand, recovering C1QBP reestablished their animal tumorigenicity in these knockdown cells.

Notably, C1QBP was also reported to be involved in glutamine oxidation (Fogal et al., 2015). It is commonly known that glutamine is essential for mammalian cell proliferation. It is therefore associated with tumor progression of any type of cancer. Higher glutamine oxidation positively correlates with a higher degree of tumor progression *in vivo*. Myc can upregulate *C1qbp* transcription, resulting in the regulation of glutamine metabolism, which suggest that a high level of Myc in malignant brain cancers is accompanied by increased expression of C1QBP. On the other hand, attenuation of C1QBP expression stunted glioma cells’ growth. C1QBP silencing in glutamine-addicted glioma cells induced resistance to glutamine deprivation. In other words, C1QBP is involved in mitochondrial metabolism and plays an integral role in Myc-induced glutamine addiction in cancer cells.

Additionally, C1QBP has been shown implicated in mitochondrial-dependent apoptosis of tumor cells. Hu et al. reported that siRNA-mediated *C1qbp* knockdown enhanced mitochondrial fragmentation, which was accompanied by a loss of detectable levels of the mitochondrial fusion mediator proteins Mfn 1/2. Furthermore, siRNA-mediated C1QBP knockdown enhanced tumor cell death in response to cisplatin treatment (Hu et al., 2013).

**FIGURE 1:**
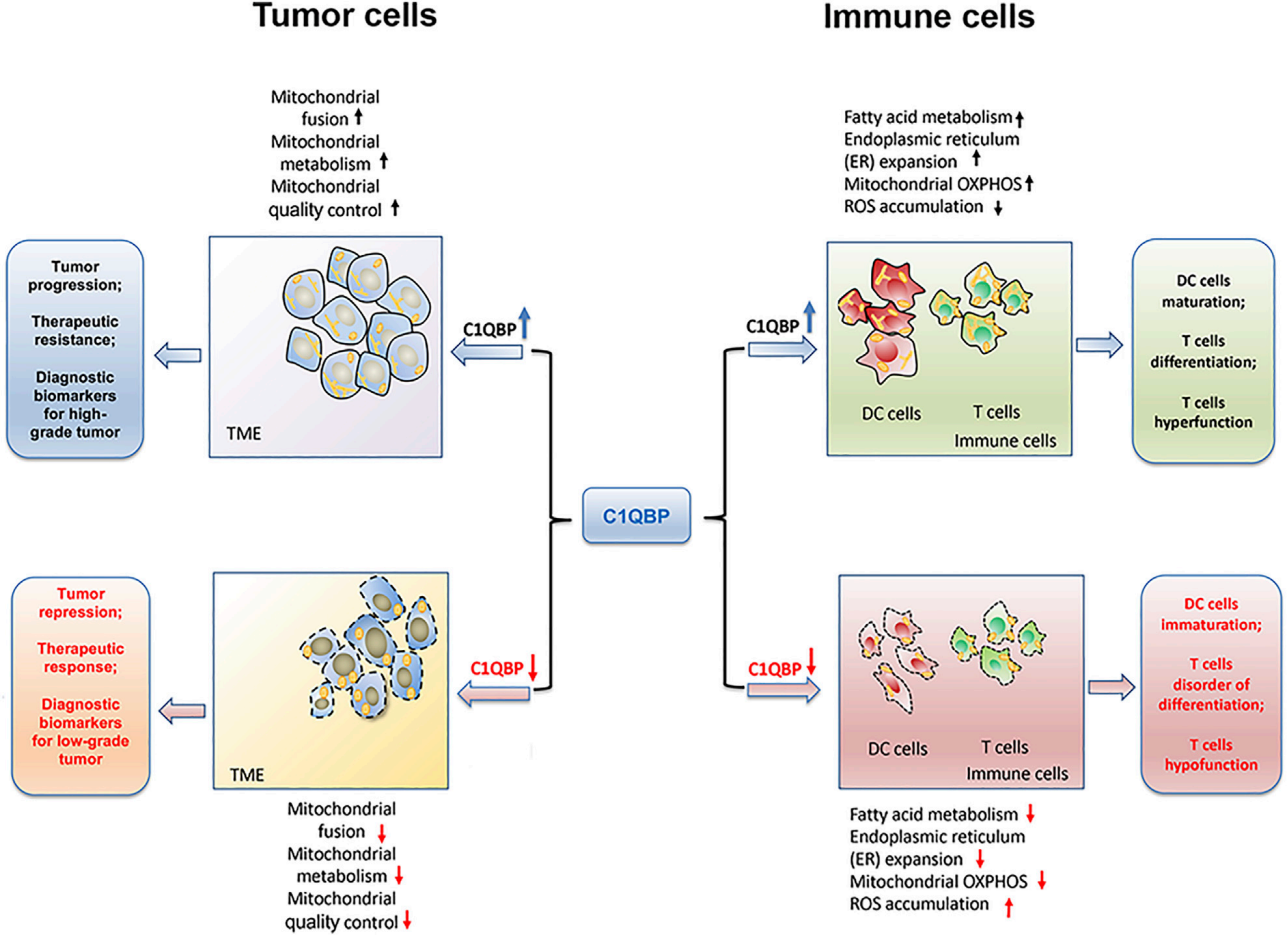
C1QBP regulation of mitochondrial plasticity to impact tumor progression and antitumor immunity. C1QBP regulates mitochondrial fusion, mitochondrial OXPHOS, and mitochondrial quality control to impact tumor malignant progression and therapeutic response. On the other hand, C1QBP also changes fatty acid metabolism, ER expansion, and ROS accumulation, thus modulating DCs’ maturation as well as T cells’ differentiation and their effector function.

Overall, C1QBP regulates mitochondrial metabolism and dynamics to potentiate mitochondrial plasticity, thereby endowing tumor cells with a bioenergetic advantage for proliferation, metastasis, and resistance to therapies, as shown in Figure 1.

## Complement C1q binding protein regulates immune cells maturation, differentiation and antitumor immune function

Warburg hypothesized that the shift to aerobic glycolysis was a cause of cancer. Aerobic glycolysis supports macromolecule synthesis, rapid proliferation and heightened ATP demand, which are necessary for tumor rapid progression. However, even though tumor cells have high rates of glucose consumption *via* glycolysis, mitochondrial function was later found to be essential in several types of tumor (Wallace, 2012; Ward and Thompson, 2012). A significant proportion of their ATP is still produced *via* OXPHOS, again suggesting that at least some mitochondrial function is preserved in tumor cells. Similar to tumor cells, immune cells always shift to glycolysis to support their rapid growth and production of biosynthetic factors for differentiation into effector cells (Kouidhi et al., 2017). However, mitochondria generate metabolic intermediates that are important for their growth and proliferation as well as for their cytotoxicity and cytokine production. Several mitochondrial energy metabolism pathways have been implicated in shaping T-cells function and differentiation (Sena et al., 2013). Besides, memory CD8^+^ T cells require mitochondria-mediated FAO to support their long-term survival (Araki et al., 2009). Therefore, it is evident that mitochondrial function not only affects tumor onset, progression and distant metastasis but also controls immune cells’ maturation, differentiation, and long-term maintenance. A body of literature indicates that C1QBP plays a vital role in mitochondrial-dependent metabolic flexibility of critical immune cells of the immune systems (Gotoh et al., 2018; Zhai et al., 2021; Tian et al., 2022).

### Complement C1q binding protein impacts dendritic cells’ maturation

Dendritic cells (DCs) have different functions, and among them are the following ones: 1) constitute the most potent antigen-presenting cells; 2) induce primary immune responses; 3) capture antigens in peripheral tissues; 4) recognize pathogen-associated molecular patterns of invading microbes post-Toll-like receptors (TLRs) ligation, which leads to cellular activation and changes in gene expression and cellular metabolism in DCs (Banchereau et al., 2000; Kawai and Akira, 2011). Gotoh et al. (2018) reported that C1QBP was indispensable in DCs metabolism and maturation. Their study found that mitochondrial C1QBP and pyruvate dehydrogenase (PDH) activity are necessary for DC maturation both *in vitro* and *in vivo*. Although pyruvate and lactate were similarly produced by both W.T. and *C1qbp* ^−/−^ DCs, after lipopolysaccharide (LPS) stimulation, LPS-induced citrate production was impaired *C1qbp* ^−/−^ DCs. Generally, glucose is processed by the cytosolic glycolytic pathway to generate pyruvate. Acetyl-coenzyme A (acetyl-CoA) is produced from pyruvate by the PDH complex and subsequently reacts with oxaloacetate to form citrate in mitochondria. Given that after LPS stimulation, the upstream metabolites of citrate in wild type (WT) and *C1qbp* ^−/−^ DCs were produced at similar levels, the authors focused on the activity of PDH in both DCs types. They revealed that C1QBP could bind to PDH-E2 in DCs by immunoprecipitation experiments. Furthermore, the PDH activity in WT was significantly higher than that in *C1qbp* ^−/−^ DCs at the basal condition, which means C1QBP may control PDH activity by binding to PDH-E2 to regulate citrate production and further support DC. maturation. Notably, PDH-mediated citrate production from pyruvate is involved in fat acid synthesis (FAS). This mechanism impacts the endoplasmic reticulum (ER) expansion. Interestingly, ER expansion was observed in WT DCs but not in *C1qbp* ^−/−^ DCs after LPS stimulation.

Therefore, C1QBP bound to PDH-E2 promoted the activity of PDH, facilitating citrate production and enhancing FAS and ER expansion. In this regard, C1QBP involvement in regulating DCs maturation by controlling mitochondrial metabolism other than the electron transport chain (ETC) has many consequences to help fine-tune mitochondria expression, and alterations could lead to the decreased immune function of DCs.

### Complement C1q binding protein is involved in T cells differentiation

Besides DCs maturation, C1QBP was also reported to be implicated in the regulation of T cells differentiation. Zhai et al. (2021) revealed that C1QBP was involved in the modulation of effector CD8^+^ T cells differentiation to affect their antiviral and antitumor immune responses further. Firstly, *C1qbp* deficiency in T cells intrinsically impaired the differentiation of effector CD8^+^ T cells, which is attributed to a failure to increase their mitochondrial respiratory capacities. They found that activated CD8^+^ T cells had greater glycolytic and OXPHOS requirements than activated CD4^+^ T cells and were more sensitive to OXPHOS impairment. In other words, C1QBP-mediated augmentation of OXPHOS was necessary for the differentiation of effector CD8^+^ T cells. Moreover, C1QBP promoted the epigenetic and transcriptional programs of effector CD8^+^ T cells. It achieved it by controlling the production of metabolites, including acetyl-CoA, fumarate, and 2-HG. Their study revealed that *C1qbp* deficiency mediated OXPHOS impairment. This, in turn, capitulated in decreased acetyl-CoA but increased fumarate and 2-HG. Moreover, downregulation of acetyl-CoA subsequently dampens histone protein H3K27 acetylation, which enhances CpG methylation to reduce some genes expression encoding several master transcription factors that drive effector CD8^+^ T cell differentiation such as *Id2, Prdm1,* and *Tbx21*.

Consequently, CD8^+^ T cell differentiation becomes retarded. C1QBP facilitates the epigenetic and transcriptional programs of effector CD8^+^ T cells differentiation by controlling metabolites coupled to OXPHOS. In this regard, they provide a mechanistic association between mitochondrial metabolism and CD8^+^ T cell differentiation, which means these metabolite supplementations or targeting OXPHOS may have significant application in T cell therapies for cancer.

### Complement C1q binding protein regulates T cells exhaustion, proliferation and anti-apoptosis

Tumor-infiltrating T cells encounter not only persistent tumor antigen but also experience metabolic stress from the tumor microenvironment such as glucose competition, metabolite accumulation, and hypoxia (Vardhana et al., 2020; Yu et al., 2020). Scharping et al. (2016) revealed that continuous antigen stimulation, coordinated with hypoxia, impaired mitochondrial functions to drive T cells terminal exhaustion. Although hypoxia alone cannot induce T cells dysfunction, its association with persistent antigen stimulation plays a fundamental role in T cells exhaustion. While on the one hand, the tumor microenvironment makes tumor-infiltrating T cells with dysfunctional mitochondria have a lower mass and activity (measured as spare respiratory capacity), driving T cells’ metabolic insufficiency. On the other hand, the continuous activation of tumor-infiltrating cells further increases mitochondrial reactive oxygen species (ROS) to exacerbate the exhausted phenotype of tumor-specific T cells. Of note, mitigating hypoxia effectively alleviates these tumor-infiltrating T cells exhaustion. Mitochondria has a fundamental role in modulating T cells’ development, fate, and function. Fine-tuning mitochondria is critical for altering the different metabolic preferences and activities within cells. Recently it was shown that impaired ATP production and accumulation of mitochondrial ROS in dysfunctional T cells within the suppressive tumor microenvironment aggravated T cells exhaustion (Li et al., 2020; Vardhana et al., 2020). However, the relationship between metabolic stress and T cell exhaustion remains still unclear.

The question of whether the mitochondrial protein C1QBP is implicated with T cells exhaustion is interesting. Our previous study found that *C1qbp* knockdown modulated T cells with the accumulation of ROS and the loss of mitochondrial membrane potential, thus impairing T cell mitochondrial fitness (Tian et al., 2022). At the same time, C1QBP insufficiency aggravated exhausted T cells. We assessed exhaustion markers, such as PD-1, Tim-3, and LAG-3 in these tumor-specific T cells. We found that these inhibitory receptors exhibited higher expression levels in tumor-infiltrating *C1qbp* ^+/−^ T cells than the corresponding WT cells. In this regard, *C1qbp* knocking down exacerbated the tumor-infiltrating T cells exhaustion, leading to a reduced antitumor immune response.

Chronic antigen stimulation drives the defective oxidative phosphorylation and promotes ROS generation in activated T cells, so *C1qbp* could dampen T cells proliferation while remaining susceptible to cellular apoptosis. To detect the underlying molecular mechanism, we noticed AKT/mTOR proliferation signaling pathway and Bcl-2 anti-apoptotic signaling pathway. Our previous results demonstrated that *C1qbp* insufficiency inhibited the AKT/mTOR signaling pathway, which conferred T cells with relatively weaker proliferation capacity (Tian et al., 2022). At the same time, *C1qbp* knocking down reduced the anti-apoptotic proteins recruitment, such as Bcl-2 and Bcl-XL, but aggravated caspase-3 activation and poly (ADP-ribose) polymerase (PARP) cleavage, which thus accelerated T cells apoptotic process. Therefore, *C1qbp* knockdown induces T cells exhaustion and pre-apoptosis but retarded their proliferation as well as antitumor immune function.

Overall, the potentiation of immune cells’ mitochondrial plasticity through C1QBP promotes fatty acid metabolism, ER expansion, and mitochondrial OXPHOS, but relieves the accumulation of ROS, which could further enhance the DCs maturation, T cells differentiation as well as the tumor-infiltration T cells durability (Figure 1 **)**.

## Manipulation of complement C1q binding protein impacts the competitive balance between tumor cells and immune cells

### Targeting complement C1q binding protein ameliorates tumor progression

C1QBP is involved in tumor progression and metastasis, making it an attractive, actionable target against cancer, bringing to the development of monoclonal antibodies (mAbs). Such therapies were successful in inhibiting tumor growth and migration (Sanchez-Martin et al., 2011). The unique upregulation of C1QBP in certain tumors, and targeting it, in various ways, in such tumors could be a valuable strategy (Yenugonda et al., 2017; Rousso-Noori et al., 2021). C1QBP can also be found at the cell surface of angiogenic endothelial cells besides tumor cells. In nature, C1QBP acts as a receptor bound by the CGKRK peptide. Based on such premises, Agemy *et al.* devised a tumor-targeted nanosystem, through which CGKRK pentapeptide delivers a proapoptotic peptide into the mitochondria of tumor blood vessel endothelial cells and tumor cells (Agemy et al., 2013). The treatment was highly effective, especially in the refractory glioblastoma mouse models, compared to other antiangiogenic therapies.

Moreover, another tumor-homing peptide, LyP-1 (CGNKRTRGC), capable of targeting the cell surface of localized C1QBP, has been used to deliver nanoparticles to breast tumors overexpressing C1QBP and has shown efficacy *in vivo* (Luo et al., 2010). However, both the anti-C1QBP antibody and the LyP-1 peptide targeted the C1QBP subset that was exposed on the cell surface. Yenugonda et al. (2017) developed C1QBP-directed therapeutics to inhibit the protein in all subcellular compartments, including mitochondria. A primary screening assay employing a glutamine-addicted glioma cell line identified a hit compound called M36, which could bind and inhibit C1QBP located on the cellular membrane and in the mitochondria. In addition, they further demonstrated that M36 impedes the growth of glioma cells and patient-derived glioma stem cells in culture due to C1QBP genetic knockdown.

In addition, antibody neutralization of C1QBP inhibits lamellipodia formation, cell migration and focal adhesion kinase activation (Kim et al., 2016). At the same time, this antigen-antibody reaction inactivates receptor tyrosine kinases (RTKs) activation in various cancer cells and prevents even angiogenesis, resulting in the decreased tumorigenesis *in vivo*. Notably, A novel tumor penetrating peptide, linTT1 (AKRGARSTA), as a tumor targeting ligand for nanoparticles (Sharma et al., 2017). C1QBP as the primary homing receptor for linTT1, is expressed on the cell surface of peritoneal carcinoma cell lines (Paasonen et al., 2016). Iron oxide nanoworms (NWs) functionalized with the linTT1 peptide in tandem with a pro-apoptotic peptide showed C1QBP-dependent cytotoxicity (Hunt et al., 2017). At the same time, the linTT1-NWs predominantly accumulates in CD31-positive blood vessels, in LYVE-1-positive lymphatic structures, and in CD11b-positive tumor macrophages, thus resulting in significant reduction of weight of peritoneal tumors and significant decrease in the number of metastatic tumor nodules. In this regard, C1QBP-directed intraperitoneal targeting of other anticancer agents may represent a potential strategy to improve their therapeutic index.

### Enhancement of complement C1q binding protein optimizes the antitumor function of immune cells

Considering that C1QBP is be specifically expressed on the surface of glioma cells, it is regarded as a suitable tumor associated antigen (TAA) for redirected chimeric antigen receptor (CAR) T cell therapy. Rousso-Noori et al. (2021) reported that C1QBP-specific CAR T cells exerted a dual antitumor and antiangiogenic therapeutic benefit in gliomas. Their study focused on designing CAR T cells targeting C1QBP positive glioma cells. They found that the CAR T cells targeting C1QBP reduced tumor vascularization and extended the overall survival of mice bearing gliomas, thus providing proof-of-principle evidence that C1QBP-CAR T cells can recognize and eliminate not only glioma cells but also tumor endothelial cells. Thus, CAR T cells targeting C1QBP may serve as a therapeutic option for glioblastoma patients through specifically recognizing and eliminating C1QBP expressing glioma cells and tumor derived endothelial cells, finally controlling tumor growth in orthotopic syngeneic and xenograft mouse models.

On the other hand, upregulation of mitochondrial plasticity may effectively reinvigorate T cell’s robust and persistent immune function. This could alter the metabolic competition between tumor cells and immune systems. Thus, it is worth noting that C1QBP modified CAR T cells’ immune function and their long-term maintenance. Our previous study demonstrated that *C1qbp* knockdown attenuated CAR T cells’ antitumor immune response (Tian et al., 2022). Specifically, we constructed the *C1qbp* ^+/−^ and *C1qbp* ^+/+^ CAR T cells targeting B7-H3. We found that *C1qbp* knocking down reduced CAR T cells’ release of IFN-γ and TNF-α cytokines, which reduced the corresponding immunotherapeutic efficacy of CAR T cells *in vivo*. In contrast, sufficient *C1qbp* potentiated CAR T cells’ mitochondrial adaptation to the relentless TME by ameliorating their exhaustion, thereby improving their durable antitumor immune function.

Since the mitochondrial function and metabolic flexibility imply the fate and function of immune cells, metabolic interventions manipulating immunity are rare. Using new proteins to achieve this purpose represents a great opportunity that remains untapped. Therefore, developing strategies for potentiation of mitochondrial-dependent metabolic flexibility could improve the efficacy of current immune-based anticancer therapy.

## Future direction

Mitochondrial plasticity endows cells with superior adaptation to the relentless microenvironment. This adaptation is regulated spatially and temporally by regulating gene expression and metabolic signatures, associated mitochondrial dynamics, metabolism, and quality control.

On the one hand, for tumor cells to survive, they are forced to remodel their signaling pathways by altering transcription, translation, and post-translational modifications to acquire phenotypic heterogeneity. Such heterogeneity, in turn, determines whether tumor cells resist environmental stress, enter dormancy or metastasize. Notably, it is imperative to know that mitochondrial plasticity plays a critical role in aiding cancer cells in acquiring metastatic traits under adverse environmental conditions. Cancer stem cells (CSCs) as a population of cells with stem cell-like properties are considered to be the root cause of tumor heterogeneity, which is also responsible for metastatic dissemination and therapeutic resistance (Clevers, 2011; Pattabiraman and Weinberg, 2014). CSCs are characterized by the increased mitochondrial mass and the elevated mitochondrial biogenesis (Luo and Wicha, 2015; Song et al., 2015; Peiris-Pages et al., 2016). In this scenario, mitochondrial biogenesis accompanied with increased membrane potential, and higher generation of mitochondria-derived ROS, has recently been regarded as a regulatory instigator of tumor stemness. Thus, the question whether C1QBP would regulate mitochondrial biogenesis to further impact tumor stemness will be of interest.

On the other hand, we must also stress that potentiation of immune cells, through enhancement of mitochondrial plasticity, contributes to preventing immune exhaustion and durable antitumor immunity. Improving T cells’ mitochondrial fitness can result in persistent immune function. It can cope with a stressful microenvironment. Consequently, in our opinion, an enhancement of mitochondrial function could be a promising strategy for tumor immunotherapy that could be used in combination with the current checkpoint blockade therapies available, such as anti-PD-L1, PD-1, and CTLA-4. Besides, a successful immune response depends on the ability of T cells not only to proliferate extensively and attain effector functions but also to generate long-lived memory T cells. FAO engagement is critical for the generation of memory T cells (Raud et al., 2018). C1QBP was reported to be involved in regulation of lipid metabolism and homeostasis (Liu et al., 2017; Liu et al., 2018). Therefore, the question whether metabolic reprogramming such as FAO through modulation of C1QBP would probably impact the generation of long-lived memory T cells requires further evaluation.

**FIGURE 2:**
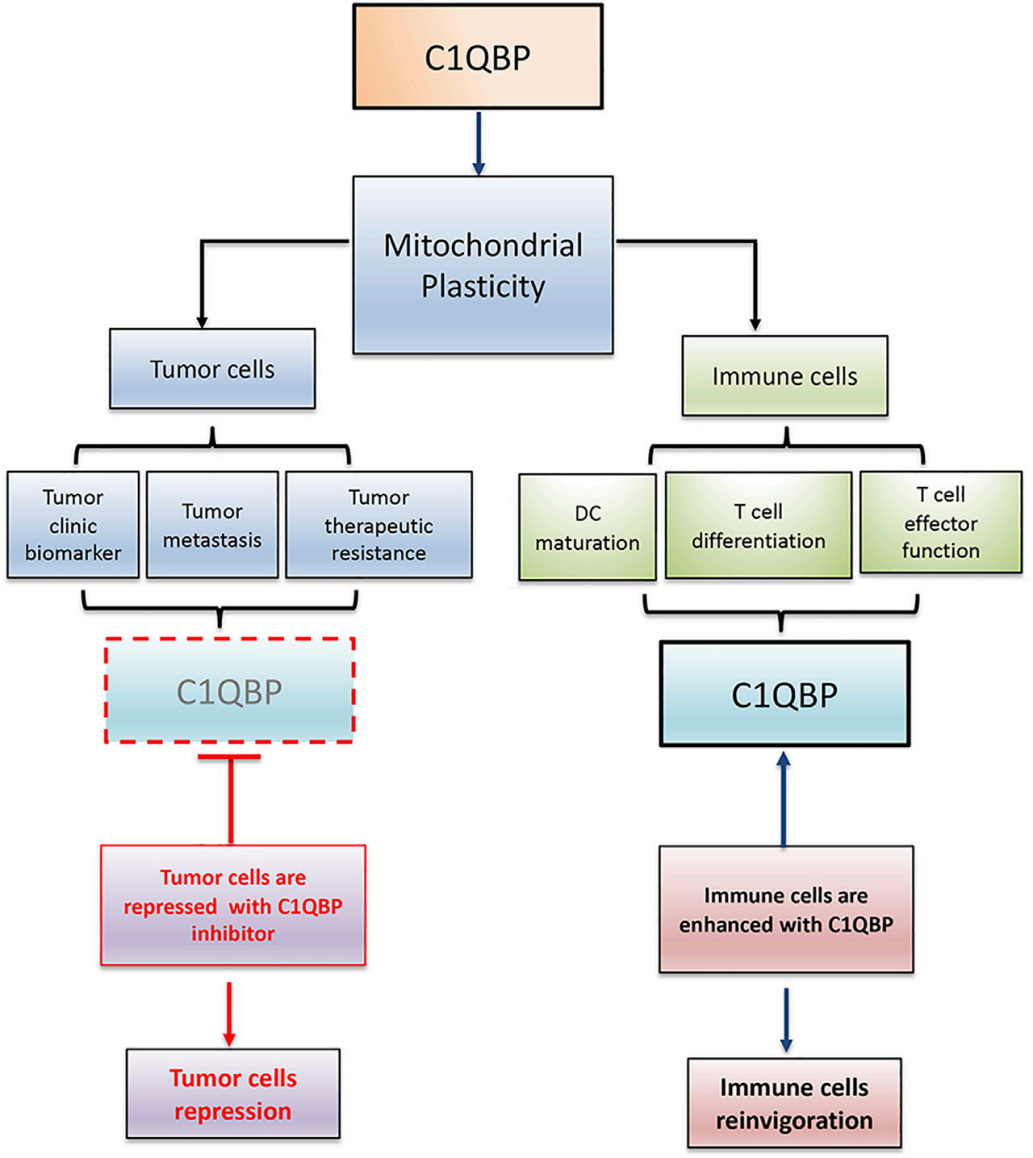
C1QBP regulation of mitochondrial plasticity alters the competitive balance between tumor cells and immune cells. Tumor cells disarmed from C1QBP exhibit reduced tumor proliferation, metastasis, and therapeutic resistance, while immune cells potentiated with C1QBP perform the active and durable antitumor immunity. In this regard, strategic manipulation of C1QBP would repress tumor progression but optimize antitumor immune function.

In conclusion, C1QBP is vital in maintaining mitochondrial plasticity. Therefore, inhibition of C1QBP in tumor cells and promotion of C1QBP in immune cells would beneficially adjust the competitive balance between tumor and immune cells, providing a very reasonable therapeutic opportunity by finding a way to correctly fine-tune mitochondrial-based metabolic flexibility (Figure 2).
